# Books Review

**Published:** 1870-09

**Authors:** 


### Books Review.

*Da Costa's Medical Diagnosis: third edition.* By J. B. LIPPENCOTT & Co., *Philadelphia,* 1870.

It is quite unnecessary for us to speak in detail of the excellencies of this work, which is now so favorably known, and thoroughly appreciated by the proession. The modestly author speaks of it as designed to furnish advanced students a guide that might be of service to them in their endeavors to discriminate disease. While it is admirably calculated to do this, it is yet, if possible, better suited to the wants of the active practitioner, neither burdened with unnecessary minuteness, or wanting in precise and practical knowledge.

The third edition is revised, extended and improved, but still the progress of the art will not permit of many important additions in the short time which has elapsed since its first appearance. This is a work which has every where been received with great favor, and has thus passed very early to third edition. It is unsurpassed in its style, matter and method, and is really as comprehensive and complete as the science of medicine will at the present time permit.

*Medicine and Morals: An Address before the Onondago Medical Society, at Syracuse.* By J. A. MOWRIS, M. D.

Dr. Mowris is fearfully abusive of the speculum, and those who use it. From his accout it does appear that Syracuse is pretty thoroughly speculated, but we think the Doctor carries his war too far. There may be dishonest Doctors who speculate for money, and for all the other purposes he mentions; but when he writes the following paragraph he shows some personal hatred, not born of love to science or humanity. We think, after reading the paper a second time, that it contains some truth, but it cannot be said to be impassionate.

" Those medical gentlemen who seem to fancy that the circulation of the speculum was the chief end of their creation, have seemed impatient to know to what extent I endorse their hobby. I would prefer to define my position on this point in a *paper* rather than in a paragraph, but that they may have no further pretext for misrepresenting me, I will briefly explain.

The Uterine *Speculum*, I believe, was invented in the interest of medical science. I *know* that there are cases in which the instrument is valuable as a means of diagnosis. In the same time concerning the *Specialty* I *as* firmly believe that its *cultivation* never was conducted by clean hands, that *practically*, if not *inherently*, it is the *child of corruption*, the most *illusive*, *insidious*, and *effective demoralizer of the present day ;* that it needlessly deflorates the virgin, favoring her ruin, or, escaping, it brings her nuptial bed under the cloud of unjust and cruel suspicion.

Aye sir, and *more* than this, it has become to the WIFE—to her who alone of all her sex, is commissioned for that consigning embrace which lights the lamp of the soul, to *her* it has become the extinguisher of maternal affection. On a former occasion I characterized it the rampant Herod of the nineteenth century. Let me now, sir, be just to the ancient dead. The jealous king came with that merciful instrument, the sword, which did *not* debauch woman—which, while indeed it laid low the innocents, left woman uncorrupted, unpolluted, untouched. For amid the desolation, thank Heaven! there yet was Rachel, in the sublimity of true motherhood, weeping for her children, refusing comfort.

Sir, the Uterine Specialty is no common evil. It subverts the foundations of civilization, and pollutes the fountains of public virtue. Its direct antagonism to the spirit and design of our high calling is too conspicuous, too flagrant to escape the reproving notice of the profession. The vital question is inevitable. It urges itself on the mind and judgment, aye, sir, and better still, on the *conscience* of every physician present.

Shall we longer suffer this scourge to devastate under the banner of our beneficent profession ? "

---

### *Fluid Extracts.*

We have received specimens of Fluid Extracts, from G. W. Hazeltine, manufacturing chemist and pharmacist, Jamestown, N. Y., which appear to be unsurpassed for purity and excellence. We believe that Jamestown and vicinity can safely and profitably rely upon home manufactures, and believe that the earth furnishes nothing better.

*The Preventive Obstacle, or Conjugal Onanism.* By L. F. E. BERGERET. Translated from the French by P. DE MARMON, M. D. TURNER & MIGNARD, 109 Nassau Street, N. Y.

This appears to be a truthful and well written book, setting forth the dangers and physical evils of sexual frauds. As this whole matter of sexual connection is now being published freely, in all forms, it is doubtless much better to make the truth available rather than shut it out, thus giving error and inconsistency the whole field.

This work is designed for the profession, but cannot fail to reach the popular eye; if so it will be an instruction and a warning.

---

*Life at Home: or the Family and its Members.* By WILLIAM AIKMAN, D. D. SAMUEL R. WELLS, New York, 1870.

We are not much accustomed to reviewing "Family Books," yet finding so much in this work to commend, and little to oppose, we conclude to say of it, that it is *first rate*, that it contains the true principles of love and good will; that it warns, instructs, encourages, strengthens, and protects the "life at home." So far as we are able to judge of a series of discourses upon marriage, husbands, wives, children, etc., etc., they are instructive, and well adapted to the family circle.

---

*On the relative action of Calomel in disease. By* FREDERICK D. LENTE., M. D., *Coldspring, N. Y.*

This is a paper read before the Dutchess County Medical Society, in January last, and consists of a very well written argument for large doses of calomel, twenty to sixty grains, frequently repeated, in the treatment of dysentery and croup. The safety and efficacy of the plan is shown by well reported cases, from which it appears that calomel is really the only reliable medicine in these and similar diseases, and is capable of affording relief after all other plans of treatment have failed. Its beneficial effects are attributed to a "sedative action this drug in large doses has upon the system."

---

*Report upon the best methods of treatment for different forms of Claft Palate. By* WM. R. WHITEHEAD, M. D.

We have perused this paper with considerable care, for many surgeons believe that Staphylorraphy is an operation so difficult to make in many cases, and in all, or nearly all, so uncertain in its results as to scarcely merit trial.

This report illustrates most beautifully the *best* methods, and is certainly very interesting and instructive; it will do something towards encouraging renewed trial.

*A Statement of the Case of the People against Elisha B. Fero.* By CHARLES H. PORTER, M.D., *Albany, N. Y.*

By this " statement," Dr. Porter makcs a very valuable contribution to medical jurisprudence. He also shows how important it is that physicians, when called as witnesses, give a rational reason for their opinions. When they turn advocates, or become partizans, they are out of their natural elements.

---

*Pathology of* BRIGHT'S *disease. By* WILLIAM B. LEWIS, M. D.

This is a most instructive and valuable lecture upon the pathology of Bright's disease, and is illustrated by wood cuts, showing the microscopic appearances very satisfactorily. The student of the microscopic pathology of venal diseases will be deeply interested in the paper; indeed all general practitioners of medicine will be instructed by a careful perusal.

---

*Physiological actionand therapeutic uses of "Acidium Phosphoricum Dilutum." By* JUDSON B. ANDREWS, M. D.

This paper is an attempt to demonstrate the physiological action of phosphoric acid, by the traces of the sphygmograph. It is an ingenious and instructive paper, and well worth perusal.

---

*Three cases of imperforate Anus, with remarks. By* J. H. POOLEY, M. D., *of Yonkers, N. Y.*

Dr. Pooley reports three very interesting cases of imperforate Anus, with the operations made for relief, which terminated favorably in two of the cases, while the third, from its nature and extent, proved fatal. His remarks upon the natnre and modes of treatment are correct and sensible.

